# Deciphering controversial results of cell proliferation on TiO_2_ nanotubes using machine learning

**DOI:** 10.1093/rb/rbab025

**Published:** 2021-06-21

**Authors:** Ziao Shen, Si Wang, Zhenyu Shen, Yufei Tang, Junbin Xu, Changjian Lin, Xun Chen, Qiaoling Huang

**Affiliations:** 1 Department of Physics, Research Institute for Biomimetics and Soft Matter, Fujian Provincial Key Laboratory for Soft Functional Materials Research, Xiamen University, Zengcuoan West Road, Siming District, Xiamen 361005, China; 2 State Key Laboratory for Physical Chemistry of Solid Surfaces, and Department of Chemistry, College of Chemistry and Chemical Engineering, Xiamen University, 422 Siming South Road, Siming District, Xiamen 361005, China; 3 Wenzhou Institute, University of Chinese Academy of Sciences, No.16 Xinsan Road, Hi-tech Industrial Park, Wenzhou, Zhejiang, 325000, China

**Keywords:** TiO_2_ nanotubes, cell proliferation, controversial results, machine learning

## Abstract

With the rapid development of biomedical sciences, contradictory results on the relationships between biological responses and material properties emerge continuously, adding to the challenge of interpreting the incomprehensible interfacial process. In the present paper, we use cell proliferation on titanium dioxide nanotubes (TNTs) as a case study and apply machine learning methodologies to decipher contradictory results in the literature. The gradient boosting decision tree model demonstrates that cell density has a higher impact on cell proliferation than other obtainable experimental features in most publications. Together with the variation of other essential features, the controversy of cell proliferation trends on various TNTs is understandable. By traversing all combinational experimental features and the corresponding forecast using an exhausted grid search strategy, we find that adjusting cell density and sterilization methods can simultaneously induce opposite cell proliferation trends on various TNTs diameter, which is further validated by experiments. This case study reveals that machine learning is a burgeoning tool in deciphering controversial results in biomedical researches, opening up an avenue to explore the structure–property relationships of biomaterials.

## Introduction

Upon intimate contact of tissue with biomaterials, spontaneous adsorption/adhesion of biological components occurs rapidly, including small molecules adsorption, protein adsorption, cell adhesion, matrix deposition, etc. [1–3]. However, this process is extremely sophisticated as it involves not only luxuriant bio-components (such as proteins, cells, blood, etc.) but also convoluted biomaterials with various properties [4–6]. With decades of endeavors, our understanding of the structure–property–function relationships grows with a common knowledge that a small change of single property could affect biological responses, leaving many impenetrable fundamental problems [7–9]. Especially, with copious controversial examples keeping recurring incessantly in the literature, the structure–property relationships remain enigmatic. For example, a general rule for cell adhesion is that hydrophilic surfaces are more conducive to cells than hydrophobic surfaces [10]. But many studies indicate hydrophobic substrate might induce better cell adhesion [11].

After scrutinizing the literature, we find that the contradictory results might be caused by different experimental details, including cell species and origins, cell density, protein types and concentration, sample variabilities, etc [12, 13]. But could we get more information about structure–property relationships from publications to improve materials design and fabrication? Is it possible to obtain ‘controversial results’ or desirable results by rational design of experimental variables, i.e. could we manipulate the results by strategical tweaks of the variables? The answer is ‘It depends’. Apparently, the current information we acquired from human knowledge, literature analysis, and massive data is incomplete. More importantly, with the rapid development of biomaterials science, it is challenging to keep pace with the growing rate of data/publications using traditional analysis methods. Thus, it is difficult to use traditional methods to solve the questions above. We need other methodologies to learn from vast literature, unravel the sophisticated relationships, and recommend experimental features for desired properties.

As an interdisciplinary subfield of computer science, machine learning uses data analysis methods to discover hidden, attractive, and potentially useful patterns and relationships from massive data [14, 15]. Compared with traditional data analysis methodologies, machine learning can abstract and build meaningful mathematical models from reality and convert extensive data into useful information and knowledge [16]. For example, Paul *et al*. employed machine-learning algorithms to train failed or unsuccessful reaction data and built models to predict conditions to form a brand-new organically templated inorganic product with a high success rate [14]. Additionally, in terms of material design, machine learning methods could save researchers from the dilemma of the traditional trial-and-error methods by establishing a model that could quantitatively describe the relationships between experimental conditions and properties [17]. For instance, the neural network is a multi-layer feedforward network trained by a backpropagation algorithm. It can provide a new recipe to realize the property-oriented compositional design for high-performance complex alloys by training the collected data from publications [16]. Herein, the advantage of machine learning in processing and analyzing a comprehensive set of literature data can be exploited to explore the structure–property relationships of biomaterials.

As a case study, we utilize machine learning algorithms to explore structure–property relationships between titanium dioxide nanotubes (TNTs) and cell responses. In the past decade, TNTs have been extensively proved to be an excellent candidate for biomedical applications, including bone/dental implants, drug delivery, cardiovascular stents, biosensors, brain tumors, etc [18–21]. However, the relationships between cell responses and nanotube diameter remain unclear. For example, Park *et al*. demonstrated that cell differentiation decreased with TNTs diameter, with 15 nm TNTs significantly accelerating cell differentiation [22]. In contrast, Yu *et al*. showed that TNTs with larger diameter (120 nm) had higher cell (canine bone marrow stromal cells) proliferation and increased gene expression (Runx2, COL1, OCN) than TNTs with diameters of 30 and 70 nm [23]. It is understandable the discrepancies derive from the synergistic of experimental parameters, including cell type/source, cell culture media components, diverse TNTs (e.g. variation in nanotube diameter, nanotube length, wall thickness, crystalline phase, etc.), and sterilization method, etc [13, 24]. Tremendous endeavors have been devoted to exploring the structure–property relationships; however, controversial results keep recurring [13, 23, 25]. It is unclear the feature importances of each experimental variable. And it is ambiguous whether we can overturn cell proliferation patterns on diverse TNTs by tuning only one variable, or does the adjustment of multiple variables required? For instance, it has been demonstrated that annealing could enhance cell proliferation [26], that we tested if annealing could tune cell proliferation patterns on diverse TNTs. However, to our surprise, annealing would not affect cell proliferation patterns on TNTs with a wide diameter range [27, 28]. Apparently, it is tedious to explore all other possibilities through traditional experiments.

In this paper, we resorted to machine learning approaches to decode the relationships and rank the feature importance via thorough learning of related publications. We used the model to traverse all kinds of possibilities and chose two sets of experimental features that were most likely to induce reverse cell proliferation patterns. Further experimental verification proved the feasibility of our model in predicting cell proliferation patterns.

## Materials and methods

### Data acquisition

We thoroughly searched relevant publications from Web of Science, Google Scholar, PubMed, NCBI, etc. We retrieved over 1000 records from all different search engines and narrowed them down to 68 publications involving cell proliferation on TiO_2_ nanotubes with different diameters. As the data quality is critical to the following machine learning step, the experimental features utilized for learning should be available in most publications to ensure the accuracy and integrity of data. By elaborative examination of the publications mentioned above, we set collected experimental features that are available in most publications, including nanotube diameter, sample annealing, sterilization method, incubation time, and cell density. For the publications that did not state the sterilization method, we set it as wet autoclaving as commonly used. As for cell density, in some researches, the cell seeding number was calculated for the 24-well plates instead of the surface area of samples. We computed cell density as cell number per square centimeter of the testing samples to maintain consistency between different publications.

In the biomaterials science field, most publications do not provide open-source datasets. So we acquired cell proliferation data from the graphs by GetData Graph Digitizer (2.26) according to the manual. To reduce the difference between different literature, we have to normalize the data. For most publications, titanium foils were set as the control. So titanium was utilized for the normalization of each publication. A database containing around 270 well-labeled samples from 29 publications was established for the following machine learning process (Fig. S1, online supplementary material).

### Data processing

The data should be preprocessed before feeding into our models. For categorical variables/features, such as annealing and sterilization, we applied one-hot encoding to improve the model's performance. One hot encoding process can convert categorical variables into a binary form which machine learning algorithms can easily recognize. It could avoid the appearance of decimals with no practical meaning during the calculating process. Then all data were normalized by Min–Max scaling using the following metric to eliminate numerical gaps between features:

(1) $$x^{'}=\frac{x-x_{\min}}{x_{\max}-x_{\min}}$$

where *x* is the original value or assigned value after one-hot encoding, *x*_max_ and *x*_min_ are the maximum and minimum values in the dataset.

For the regression, the property *y* was normalized by the following metric:

(2) $$y=\frac{y_{i}}{y_{T_{i}}}$$

where *y_i_* is the original data of cell proliferation on TiO_2_ nanotubes, $y_{T_{i}}$ is the cell proliferation rate on the titanium surface.

### Computational modeling

We split the datasets into training (80%) and test datasets (20%). The training dataset was used for model building and the test dataset was used for model evaluation. As the data size was relatively small in this study (Data set in online supplementary material), we compared several models that were suitable for our dataset, including group vector machines (GVMs), support vector machines (SVM), decision Tree (DT), random forests (RF), eXtreme gradient boosting (XGBoost), and gradient boosting decision tree (GBDT) (more details can be found in online supplementary material). The R Squared (R^2^), explained variance score (EVS), mean absolute error (MAE) and mean squared error (MSE) were used to evaluate model performance. As GBDT presented higher EVS and R^2^, and lower MAE and MSE, it was used for further study. For the experimental validation, we utilized the grid search method to acquire certain experimental features for controversial results basing on the predictive ability of the GBDT model. Detailed parameters for the grid search method could be found in Table S2 (online supplementary material). After thorough exploration, we obtained two sets of experimental features (Table 1) that had the highest possibility to generate reversed results. Those two sets of experimental features were applied for the following experimental validation.

**Table 1.** Proposed experimental parameters for experimental verification

Trend	Sterilization method	Density (cells/cm^2^)	Diameter (nm)	Annealing	Time
Decreasing	UV irradiation	10 000	30, 50, 70, 100	True	1 day, 3 days
Increasing	Wet autoclaving	16 000			

### Sample preparation

The typical electrochemical anodization process was employed to fabricate TiO_2_ nanotubes with different dimensions [17]. Briefly, titanium foils of 0.1 mm thickness (99.6% purity) were cleaned by the ultrasonic cleaner in the sequence of acetone, ethanol and deionized water. The electrolyte consisted of 0.5 w/v% hydrofluoric acid in water. A platinum electrode with a size of 4 cm × 3 cm was utilized as the counter electrode. The anodization was carried at different voltages (5, 10, 15, 20 V) for 30 min before rinsing with D.I. water. The as-prepared nanotubes were further annealed at 500°C for 2 h to obtain crystalline phases. The morphologies of TiO_2_ nanotube arrays were examined by a scanning electron microscope (SEM). Crystalline phases were determined by X-ray diffraction spectroscopy (Philips, Panalytical X’pert) with Cu Kα radiation.

Two kinds of sterilization methods were employed: ultraviolet (UV) irradiation and wet autoclaving. Wet autoclaving was carried out in an autoclave sterilizer at 121°C, followed by drying at 80°C for 30 min. For UV sterilization, samples were irradiated by a UV lamp for 30 min before use. Ti foils were used as a control group for each method.

### Cell adhesion and proliferation

Mouse osteoblast-like cells (MC3T3-E1) were maintained in T-25 flasks (Corning, TCPS) placed in a CO_2_ incubator. Cells were fed with alpha-modified minimum essential medium (α-MEM-Hyclone) containing 10% fetal bovine serum (FBS, Pansera ES) and 1% penicillin–streptomycin. At the confluence of 80% to 100%, cells were harvested and seeded on the autoclaved samples with a density of 1.6 × 10^4^ cells/cm^2^, whereas a density of 1.0 × 10^4^ cells/cm^2^ was applied for UV-irradiated samples. For wet autoclaving, samples became hydrophobic that cell culture media was difficult to stay or spread out on the surface. So we utilized an O-ring on each substrate to maintain the media. Cell adhesion and proliferation were assessed after 1 day and 3 days’ culture using both fluorescence imaging (Calcein-AM, Sigma) and the WST-1 assay (Beoytime) following the manufacturer’s guidelines.

### Statistical analysis

At least three replicates were tested, and the results were expressed as a mean ± standard deviation (SD). Statistical analysis was evaluated using a one-way analysis of variance (ANOVA) followed by Turkey *post hoc* tests. A *P* value <0.05 indicates a statistically significant difference, and *P*-value <0.01 shows a highly significant difference.

## Results and discussion

### An overview of the learning strategy

**Figure 1.**
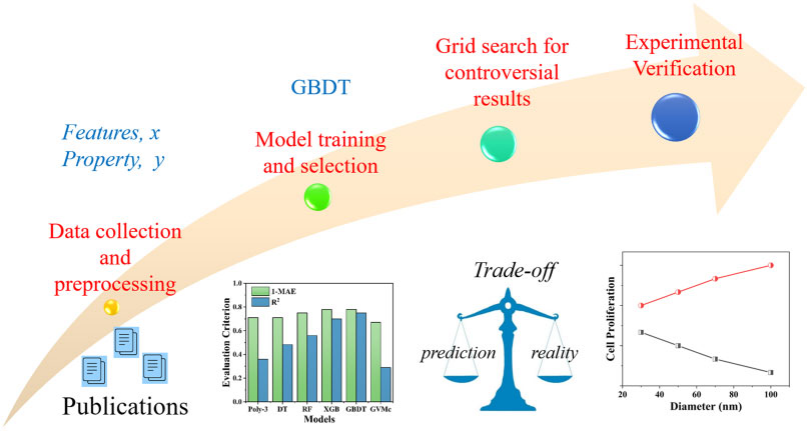
Flow chart of data-driven exploration of the relationship between TiO_2_ nanotubes (TNTs) and cell proliferation.

Figure 1 illustrates our learning strategy and shows the critical steps to explore the relationships between cell proliferation and experimental features. Generally, the learning process contains four essential steps: (i) data preprocessing and feature extraction from literature to build data set, (ii) model training and model-validation, (iii) analysis of feature importance and grid search for converse results, and (iv) assessment and validation by experiments.

### Data collection and preprocessing

The first step is to find related publications in the literature. By a thorough literature review, we retrieve 68 publications regarding cell proliferation on TNTs with varied nanotube diameters. In addition to nanotube diameter, other predominant surface properties and experimental details influencing cell proliferation include annealing, surface chemistry, surface charge, cell type, cell density, cell culture nutrients, sterilization methods, etc [13].

However, different research focuses on particular goals, and only specific details are revealed that the available data/features are uneven, and lots of essential elements are missing in some publications. It is well established that the quality of the original dataset has a significant impact on the accuracy of the resulting model. Some experimental features, such as cell type and cell passage, have been excluded from our model because similar proliferation patterns have been reported from different labs using different cell types [25, 29]. And it is impossible to track and compare the individual differences from one publication to the other. Herein, we choose features widely considered dominant factors affecting cell fate and are available in most publications, including nanotube diameter, annealing, sterilization method, incubation time, and cell density. As for crystalline phases, pure rutile or anatase TNTs are difficult to obtain, and anatase dominated TNTs are more common [30, 31]. Herein, we classify TNTs by whether annealing is applied, i.e. as-prepared TNTs vs. annealed TNTs. After a meticulous screening of all related publications, we extracted data by GetData Graph Digitizer, and our dataset is composed of 272 items screened from 30 publications [13, 18, 22–25, 29, 32–54].

### Model training and selection

Once the dataset has been built, we feed the data into the machine learning regression models for model training. Among all models selected in Section Computational modeling, the GBDT regression model has the highest coefficient of determination R^2^ score of 0.75 and the lowest Mean Absolute Error (MAE) of 0.22 on test data (other metrics are listed in the online supplementary material, Figs. S2, S3, and Table S1).

**Figure 2.**
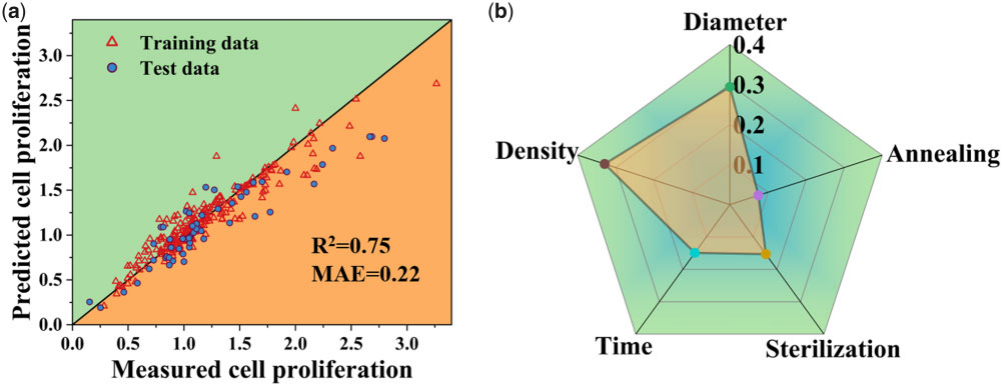
(**a**) A Comparison of GBDT predicted and measured (collected data from literature) cell proliferation values, using a split of 80% and 20% for the training and testing data. (**b**) Radar plot of the importance of each experimental feature. Diameter stands for the average diameter of TNTs, density represents cell seeding density on the samples, annealing differentiates whether samples have been annealed at high temperature, time means cell incubation time, and sterilization denotes sample sterilization methods.

Figure 2a compares the predicted values and the experimental results collected from the literature to show the GBDT model's accuracy. Most data points distribute around the diagonal, while some larger values deviate from the diagonal line. This variation is most likely attributed to the complexity of biological experiments and limited datasets, and particularly we only have a few samples of high cell proliferation values (Fig. 2a). Apparently, the model accuracy is less than satisfactory. But during model verification of specific researches, we find that the predicted cell proliferation trends are generally analogous to the reported publications (See online supplementary material, Figs. S4 and S5). Given we have limited datasets and the biases in biological experiments are high, we use the GBDT model for subsequent research and verify if we could obtain reversed cell proliferation patterns through grid search.

As an ensemble model based on the decision tree, the GBDT model can provide feature importance. The feature importance in Fig. 2b shows that cell density has the highest impact (0.33) on cell proliferation, followed by nanotube diameter (0.29), sterilization method (0.15), incubation time (0.15), and annealing (0.08). The feature importance of annealing is relatively low, but it is consistent with previous studies from our group and other groups demonstrating that annealing would not significantly affect cell proliferation on TNTs [28, 55].

### Grid search for converse results

**Figure 3.**
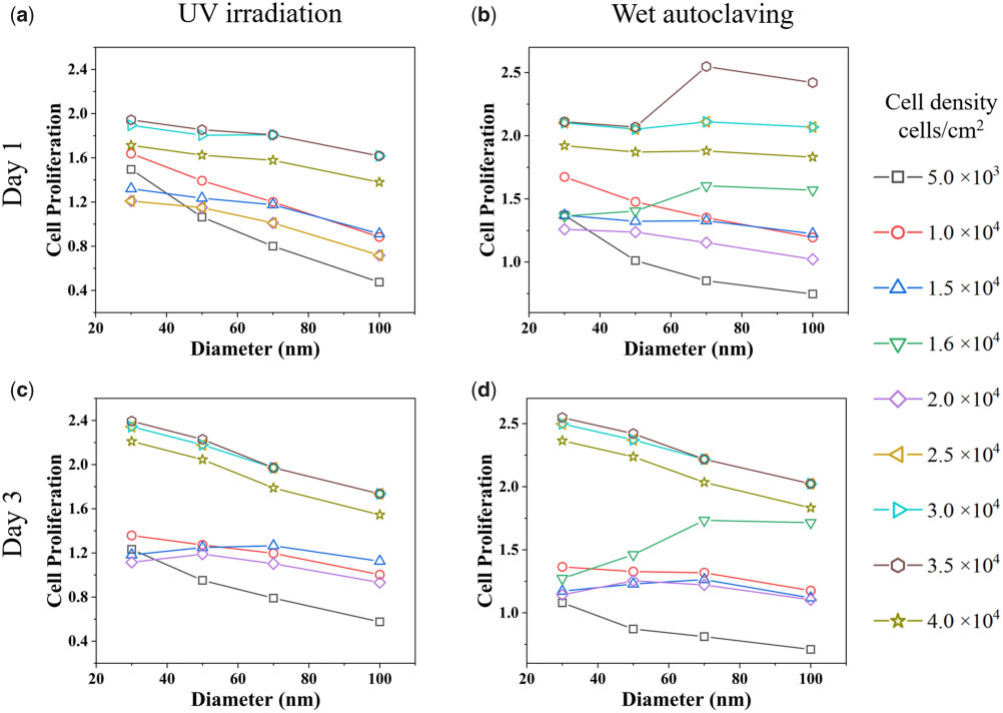
GBDT Model predicted cell proliferation trends with varied cell densities. All TNTs are annealed. Sterilization methods are UV irradiation (**a, c**) and wet autoclaving (**b, d**).

To further verify the model by experiments, we apply the grid search method to find certain experimental features to obtain controversial results (i.e. cell proliferation increases with nanotube diameter vs. cell proliferation decreases with nanotube diameter). Here, we present partial predicted data to illustrate how single features affect the results and how we choose the experimental verification features. As cell density stands out in the earlier discussion, we display how cell density affects cell proliferation on varied TNTs in Fig. 3.

When using UV irradiation on annealed nanotubes, cell proliferation decreases with nanotube diameter within a wide range of cell density. Strikingly, when using the wet autoclave method on annealed nanotubes, cell proliferation trend changes from decreasing to smooth or even increasing with the increment of tube diameter (Fig. 3b and d). We also explored if we could obtain strikingly different results by tuning other single variables (sterilization methods in Fig. S6 and crystalline phases in Fig. S7, online supplementary material), however, there is no such distinct difference. This further validates the feature importance in Fig. 2b, in which cell density has the highest feature importance, whereas the feature importance of sterilization and annealing are relatively low.

Figure 3b shows a distinct increment on 70 nm TNTs when we set cell density at 1.6$\times 10^{4}$ and 3.5$\times 10^{4}$ cells/cm^2^ on day 1, using wet autoclaving as the sterilization method. However, on day 3, the increasing pattern persists only when cell density is 1.6$\times 10^{4}\;$cells/cm^2^ (Fig. 3d). Thus, we enlist this particular combination of features for experimental verification (Table 1). As for the decreasing pattern, it seems as if cell density 5$\times 10^{3}\;$cells/cm^2^ could be a good choice. Nonetheless, such a low cell density is usually around or beyond the detection limit when using WST-1 cell proliferation assay kit for normal sample size (1 × 1 cm^2^). On the other hand, we previously demonstrated cell proliferation diminished with nanotube diameter when using the UV irradiation method [28, 56], consistent with Fig. 3a and b. Herein, we adapted experimental parameters from our previous study for the decreasing pattern [56]. Table 1 lists two sets of experimental parameters utilized for conversed results. The sterilization method and cell density are different, whereas the preparation procedure of TNT samples and cell culture time are identical.

### Experimental verification

**Figure 4.**
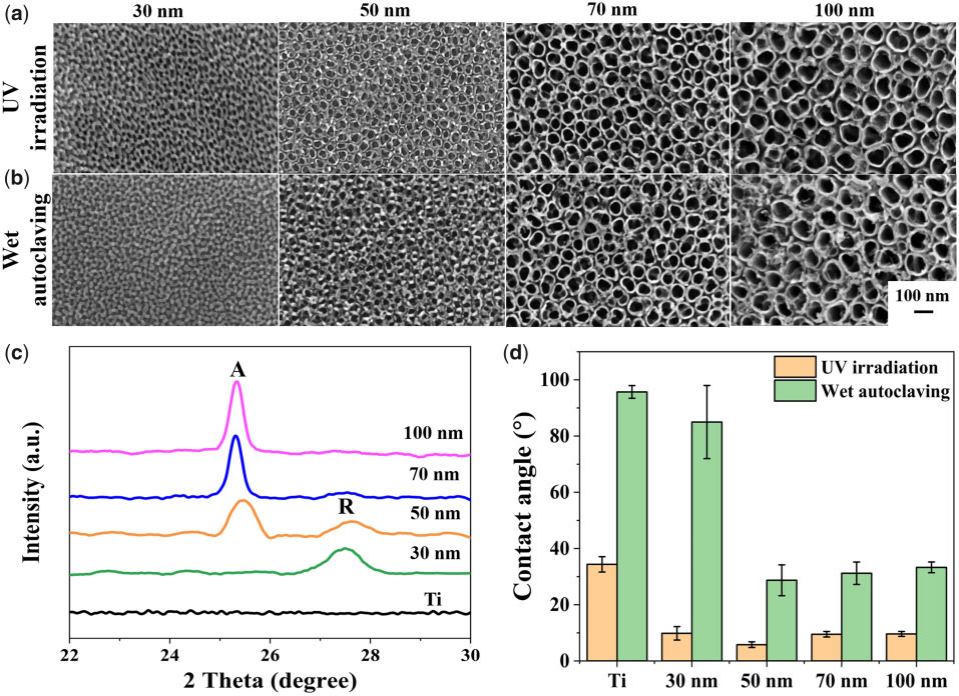
SEM Top-view of various annealed TNTs sterilized by UV-irradiation (**a**) and wet autoclaving (**b**). (**c**) XRD patterns of the annealed TNTs. ‘a’ presents anatase phase, and ‘R’ denotes rutile phase. (**d**) Static water contact angles of specimens measured after sterilization.

Figure 4a and b shows representative top-view scanning electron microscope (SEM) images of highly organized TNTs with diverse nanotube diameters. The outer nanotube diameters for the UV irradiated TNTs are around ∼30, 50, 70 and 100 nm, respectively (Fig. 4a). The tube thickness increases from ∼92 nm to 351 nm (See online supplementary material, Fig. S8). For smaller nanotubes with diameters of 30 and 50 nm, nanotube walls are thickened by wet autoclave that the wall thickness rises from 11 nm to 14 nm (See online supplementary material, Fig. S9).

The crystalline phases of the annealed TNTs are evaluated by XRD, as displayed in Fig. 4c. The peak appeared at 25.3° is identified as anatase phase (A), and the peak that emerged at 27.5° corresponds to the rutile phase (R). The smallest 30 nm TNTs are mainly in rutile form, whereas the large nanotubes with diameters of 70 nm and 100 nm are primarily in anatase form, consistent with our previous study [57]. For TNTs with a tube diameter of 50 nm, both anatase and rutile peaks appear, implying a mixture of anatase and rutile phases.

The static water contact angle is measured to detect wettability, as depicted in Fig. 4d. All UV-irradiated TNTs have contact angles smaller than 90°, suggesting the hydrophilicity of the TNTs. For UV-irradiated samples, the water contact angle decreases from 34.4° on titanium to about 10° for all TNTs with varied sizes, implying the excellent hydrophilicity of TNTs after UV irradiation. On the contrary, the autoclave method dramatically increases the hydrophobicity that the contact angles of titanium and 30 nm TNTs boost to 95.7°± 2.3° and 85.0°± 13.0°, respectively. And the water contact angles of TNTs with a diameter range of 50–100 nm raise to about ∼30°, suggesting autoclave sterilization can enhance the hydrophobicity of TNTs.

**Figure 5.**
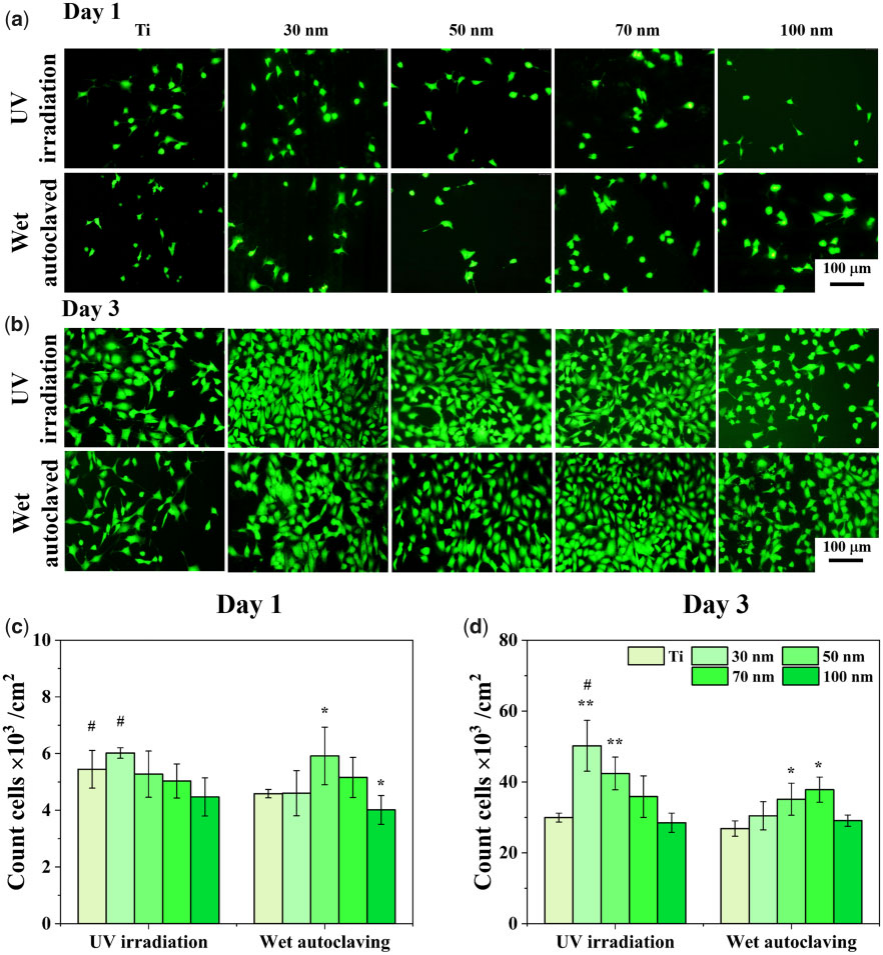
Experimental verification of model inference and design. Fluorescence images of Calcein-AM stained MC3T3-E1 cells on annealed TNTs after 1 day (**a**) and 3 days (**b**) culture. (c) quantitative statistics of fluorescent adherent cells on various samples. Unmodified titanium foils are used as a control group. *P < 0.05 indicates a significant difference comparing to titanium, and #*P* < 0.05 indicates a significant difference between UV irradiated and wet autoclaved samples.

Cell proliferation on various TNTs is evaluated by fluorescence (Fig. 5) and WST-1 (Fig. S10, online supplementary material). When UV sterilization and relatively low cell density (1.0×$10^{4}$ cells/cm^2^) are applied, cell proliferation slightly decreases with the increasing diameter on day 1, which is generally consistent with previously published results [28, 56]. In contrast, when wet autoclaving and high cell density (1.6×$10^{4}$ cells/cm^2^) are applied, a slight increase followed by a decrease could be observed on day 1, with the 50 nm TNTs having the highest cell proliferation rate. There is no distinct morphology difference in those samples, with some cells in a spherical shape and some in a fusiform shape.

On day 3, cell numbers are highly enhanced on all samples, with most attached cells in fusiform shape. A sharp decreasing pattern could be observed for UV irradiated samples. For wet autoclaving, the 50 nm and 70 nm TNTs have a higher cell proliferation rate. The WST-1 cell viability results in Fig. S10 (online supplementary material) further confirm the fluorescence results. In general, those proliferation trends are in line with the prediction in Fig. 4, demonstrating our model's effectiveness in predicting cell proliferation trends. It also indicates that we can utilize this model to predict cell proliferation and guide material design and fabrication.

On the other hand, even though cell density seeded on autoclaved TNTs is 1.6 times of that on UV irradiated TNTs, cell proliferation values on autoclaved TNTs are no higher than corresponding TNTs. And it is worth noting that cell numbers on small UV-irradiated 30 nm TNTs are statistically higher than that of autoclaved TNTs. It indicates that sterilization methods have a high impact on cell adhesion and proliferation on TNTs, and UV irradiation is more beneficial to cell adhesion and proliferation [24]. This is probably due to the changes in surface wettability during wet autoclaving, which have widely been proved to affect cell behaviors [58].

Overall, the complex and incomplete data from the biomaterials field are probably difficult to be used for machine learning study for specific quantitative predictions. However, given we could barely obtain identical results from cell-related experiments, the machine learning algorithms have excellent performance in predicting cell proliferation trends on TNTs with various dimensions.

On the other hand, it is well known that a single change in material properties will induce the difference in cell responses; however, the role played by cell density is often ignored. When cell density is low, cells could not form an essential connection, which is pivotal for cell growth. In contrast, when cell density is too high, the surface might be too crowded with cells that the difference among different biomaterials will be covered. Within an appropriate cell density range, we can tune other features (e.g. sterilization method, surface morphology, surface chemistry, etc.) to obtain different results. So it is crucial to balance cell density when exploring the structure–property effect of biomaterials.

## Conclusion

In summary, we utilize machine learning algorithms to unravel the controversial results of cell proliferation patterns on varied TNTs. We compare several algorithms, and the GBDT model reveals the best performance that we employ the model for further studies. Model validation shows the low quantitative prediction accuracy of GBDT model, however, the model performs well enough for predicting proliferation trend. Through model training, model prediction, and analysis, we acquire two sets of features that can induce the opposite cell proliferation trends further verified by experiments. GBDT model analysis suggests that cell density has higher feature importance over other features, which is further proved by experiments. More importantly, experimental verification proves that two different cell proliferation patterns could be obtained on annealed TNTs, demonstrating the efficacious of GBDT model in predicting proliferation trends. We envision that machine learning algorithms can serve as an effective tool to interpret controversial results and provide prospective insights for biomedical researches.

On the other hand, it is worth noting that the validity of machine learning algorithms relies on the dataset's size and accuracy. The biomaterials' surface interface involves too many factors, and most of them are missing in the vast literature. Hence, it remains challenging to utilize machine learning methodologies to analyze the structure–property relationships of biomaterials. Moreover, data collection from the literature should be improved by data mining approaches to improve data collecting efficiency.

## Supplementary data

Supplementary data are available at *REGBIO* online.

*Conflict of interest statement*. The authors declare no conflict of interest.

## Supplementary Material

rbab025_Supplementary_Data
