# Supplementary material for: Deciphering controversial results of cell proliferation on TiO2 nanotubes using machine learning
Source: Regen Biomater. 2021 Jun 21;8(4):rbab025. doi: 10.1093/rb/rbab025 (PMC8218935; doi:10.1093/rb/rbab025)
Supplement: rbab025_Supplementary_Data [file rbab025_supplementary_data.docx]

# Supporting Information

**Deciphering controversial results of cell proliferation on TiO_2_ nanotubes using machine learning**

*Ziao Shen, Si Wang, Zhenyu Shen, Yufei Tang, Junbin Xu, Changjian Lin, Xun Chen**, *and Qiaoling Huang**

Z. Shen, S. Wang, Z. Shen, Y. Tang, J. Xu, Dr. Q. Huang

Department of Physics, Research Institute for Biomimetics and Soft Matter, Fujian Provincial Key Laboratory for Soft Functional Materials Research, Xiamen University

Xiamen 361005 (China)
E-mail: [qlhuang@xmu.edu.cn](mailto:qlhuang@xmu.edu.cn)

Dr. C. Lin

State Key Laboratory for Physical Chemistry of Solid Surfaces, and Department of Chemistry, College of Chemistry and Chemical Engineering, Xiamen University, Xiamen 361005 (China)

Dr. X. Chen

Wenzhou Institute, University of Chinese Academy of Sciences, Wenzhou 325000 (China)

E-mail: [chenxun@ucas.edu.cn](mailto:chenxun@ucas.edu.cn), [cx8508@gmail.com](mailto:cx8508@gmail.com)

# Dataset

The dataset is visualized in three-dimensional space in Figure S1, which is constituted by 272 samples screened from published researches. The data is approximately evenly distributed between as-prepared amorphous form (123 items) and annealed crystal forms (149 items). A large proportion of the publications employ wet autoclaved (125 items) and UV irradiation (96 items) for sterilization, while dry autoclaved (37 items) and ethanol (36 items) are less used.


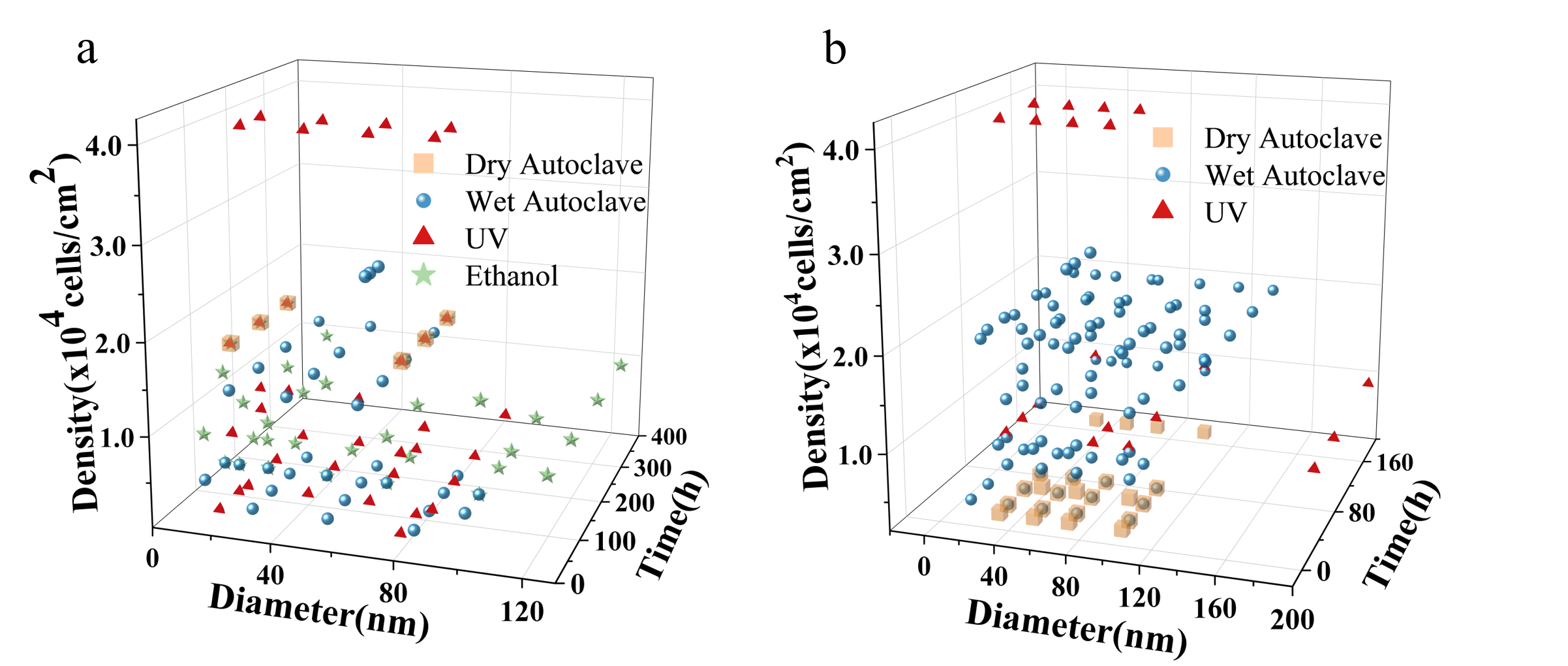


**Fig. S1.** Three-dimensional maps presenting data distribution: (a) as-prepared TNTs, (b) annealed TNTs.

# Regression models

We selected the following six classification models that are suitable for small datasets [1], based on the logic of "linear model, nonlinear model, integrated model and neural network":

1. SVM.Poly represents support vector machine with the polynomial kernel function.

$${min}_{w,b} \frac{1}{2}\left\| w \right\|^{2} (1)$$

Subjected to

$y_{i}\left( w^{T}x_{i}+b \right)\geq1, i=1,2,\ldots, m (2)$

$$K\left( x,y \right)={(\gamma\left( x,x^{'} \right)+r)}^{d} (3)$$

$\gamma={{(2\sigma}^{2})}^{-1} (4)$

where *d* is specified by parameter degree, and *r* by coef0.

1. DT: The decision tree is a model that can predict the category of a target variable through learning simple decision rules deduced from the data features.

$Gini\left( D \right)=1-\sum_{i=1}^{n} {p_{i}}^{2} \left( 5 \right)$

$$\Delta Gini\left( X \right)=Gini\left( D \right)-Gini\left( X \right) \left( 6 \right)$$

Where $p_{i}$ is the proportion of the current attributed in the data set.

1. RF: Random Forest, an ensemble algorithm that integrates votes from multiple decision trees. The traditional decision tree selects an optimal attribute of the current node when selecting the partition attribute. However, in RF algorithm, it is necessary to choose a subset containing k attributes from the attribute set of the current node before selecting the most optimal attribute from this subset to divide the dataset. The degree of randomness is controlled by k of which the recommended value is

$$k=\log_{2} d (7)$$

2. XGB: XGBoost, the “extreme gradient boosting” algorithm, which can be regarded as an improved version of the original GBDT method. Compared to GBDT, XGBoost also builds the whole model in a boosting manner by continuously adding trees and performing feature splitting to grow a tree, with several improvements. It explicitly adds regularization terms to prevent overfitting and improve the generalization ability of the model. It also introduces the second-order Taylor expansion of the loss function, which enables the customization of the loss function. Besides, XGBoost supports parallelized training, which could significantly reduce the training time on distributed systems.
3. GBDT: gradient boosted decision tree (combines gradient-descent and boosting on the training data). In each iteration, a weak classifier is trained on the basis of the residual error of the previous classifiers. The weak classifiers are models with low variance and high bias because the training process aims to improve the final classifier's accuracy by decreasing the bias. Weak classifiers are generally adopted as CART tree (i.e., classification regression tree) with small depth.

$$h_{0}\left( x \right)={argmin}_{c}\sum_{i=1}^{m} L\left( y_{i},c \right) (8)$$

$$H\left( x \right)=h_{0}\left( x \right)+\sum_{t=1}^{T} \sum_{j=1}^{J} c_{tj}I(x\in R_{tj}) (9)$$

Where $L$ is the loss function, $T$ is the number of iterations, $J$ is the number of leaf nodes and $c_{tj}$ is the best prediction in each leaf node.

1. GVMc: It is a kind of neural network based on the Monte Carlo method. It keeps approximating the function value by discarding points higher than the expected value to obtain the final result. (See attachment gvmc-regression)

Figure S2 demonstrates the difference between the predicted values and the preprocessed data from the literature. Apparently, the data points stay closer to the diagonal for the GBDT model, indicating it has a better fitting. Furthermore, the following metrics are applied to evaluate the accuracy of our model:

$$R^{2}=1-\frac{\sum_{i=1}^{n} {(y_{i}-f_{i})}^{2}}{\sum_{i=1}^{n} \left( y_{i}-\bar{y}_{i} \right)^{2}} (10)$$

$$EVS=1-\frac{var\left\{ y-f_{i} \right\}}{var\left\{ y \right\}} (11)$$

$MAE=\frac{1}{n}\sum_{1}^{n} \left| y_{i}-f_{i} \right| (12)$

$MSE=\frac{1}{n}\sum_{1}^{n} {(y_{i}-f_{i})}^{2} \left( 13 \right)$

Where n is the total number of the samples, $f_{i}$ is the predicted value, $y_{i}$ is the ground truth,$\bar{y}$ is the mean of the predicted values, and $var$ is Variance.


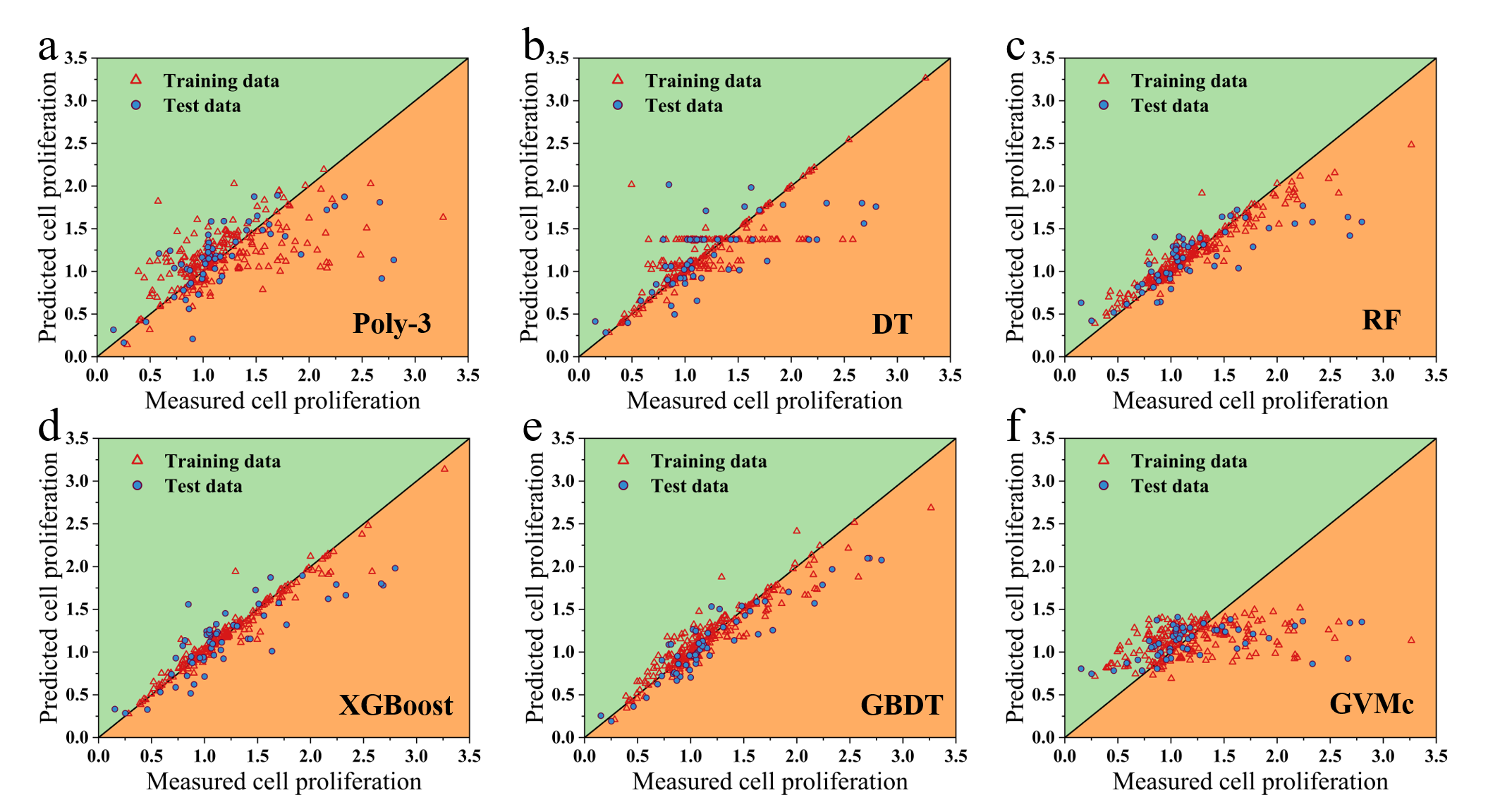


**Fig. S2.** Diagrams comparing the predicted and measured cell proliferation from different models.

**Table S1** The scores of evaluation metrics for each model.

|  | Poly3 | DT | RF | XGB | GBDT | GVMc |
| --- | --- | --- | --- | --- | --- | --- |
| EVS | 0.38 | 0.49 | 0.57 | 0.72 | 0.77 | 0.29 |
| R^2^ | 0.36 | 0.48 | 0.56 | 0.7 | 0.75 | 0.29 |
| MAE | 0.29 | 0.29 | 0.25 | 0.22 | 0.22 | 0.33 |
| MSE | 0.19 | 0.16 | 0.13 | 0.094 | 0.078 | 0.19 |





**Fig. S3.** A comparison of R^2^ and MAE among different models.

**Table S2** Parameters for the grid search method.

| Features | Values for grid research |
| --- | --- |
| Diameter (nm) | 30 , 50 , 70 , 100 |
| Annealing | Yes or No |
| Sterilization | Wet autoclaving, Dry autoclaving, UV, Ethanol |
| Time (h) | 24, 48, 72, 96, 168 |
| Density (cells/cm^2^) | 5000, 10000 - 20000 (step = 2000),  20000 - 50000 (step = 5000) |

To verify the accuracy and generalization ability of the GBDT model in predicting cell proliferation trends, we compare predictions with experimental data from the literature, i.e., we use the GBDT model to predict cell proliferation values (y, Equation 2 in Materials and Methods) of some particular cases from the literature using the same experimental parameters. We choose two sets of data: one set of data represents cell proliferation decreasing with increasing dimension, while the other set represents the opposite. It should be noted that the nanotubes utilized in different publications had varied diameters, adding to the difficulty of analysis. Thus, we replenish the model prediction with popular dimensions, including 30 nm, 50 nm, 70 nm, and 100 nm.

Figures S4a and c depict increasing trends of cell proliferation on TNTs with different dimensions from Yu et al [2, 3], and Figures S4b and d are corresponding predictions from the GBDT model using essentially the same experimental parameters in Figures S4a and c, respectively. The difference between Figure S4a and the original graph in the literature comes from data preprocessing, diminishing the discrepancy between different publications. Also, it is worth mention that all data have been normalized with the value of titanium (control) from the same day that the diagrams represent only the general rules of how nanotube diameter affects cell responses. Figure S4a shows that the cell proliferation rate increases with the nanotube dimension and reaches the highest on the largest diameter nanotube (120 nm) on day 1 and day 2. However, this rule diminishes on day 4 that there is no significant difference among different dimensions. The prediction values in Figure S4b are systematically lower than normalized experimental values in Figure S4a, reflecting the unsatisfactory of the model. However, the prediction trends in Figure S4b are analogous to that of Figure S4a, except a sudden drop is observed for TNTs with a diameter of 100 nm. This fluctuation is caused by the relatively low cell proliferation value of 100 nm nanotubes in the dataset.


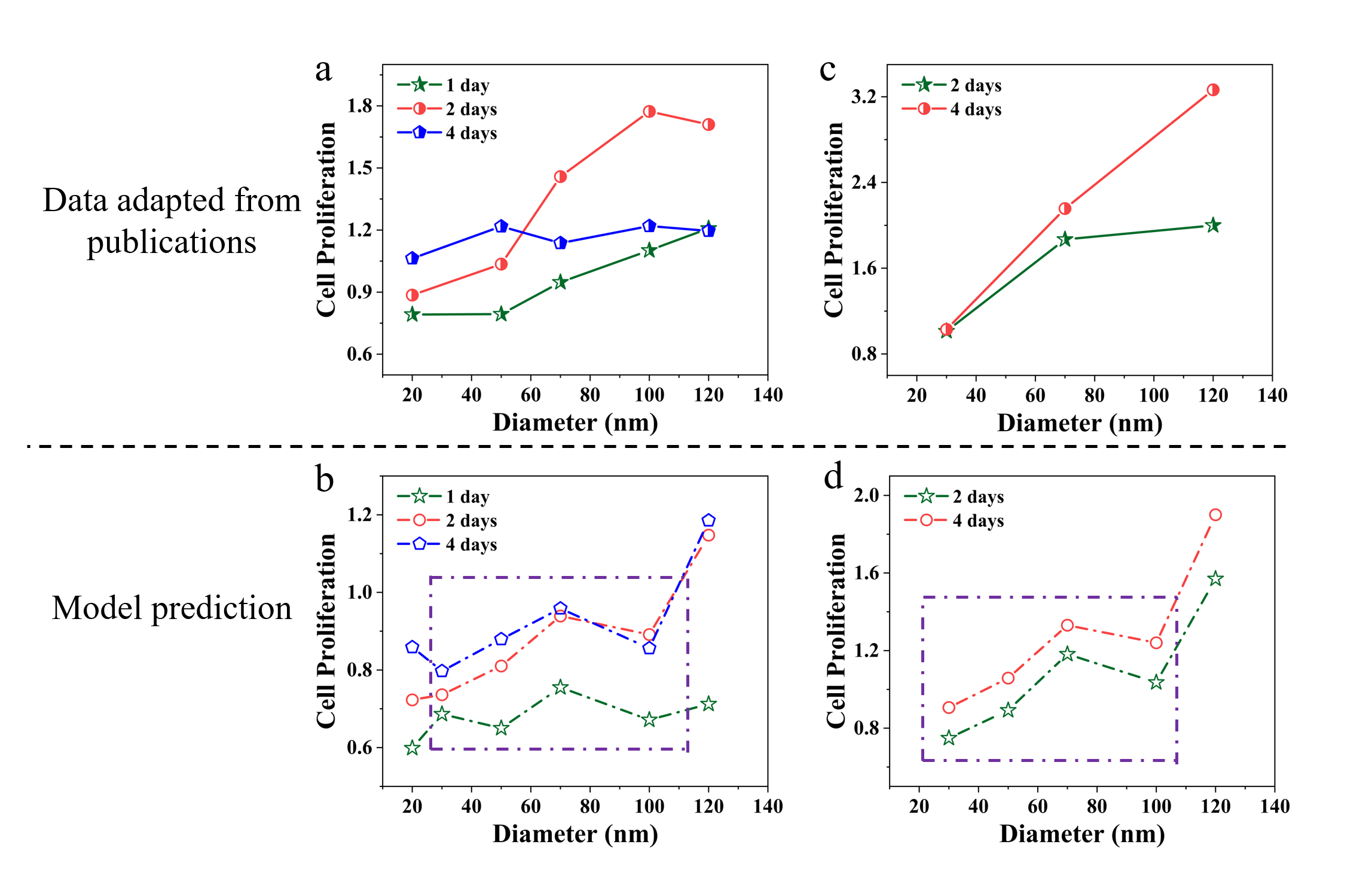


**Fig. S4.** Comparisons between measured (a and c) and predicted (b and d) values. The data inside the rectangle area represents the most popular TNTs used in the literature, illustrating a false conclusion could be drawn when using specific TNTs. Annealing and wet autoclaving are used for all figures. Cell density: 2.1×10^4^ cells/cm^2^ in (a) and (b), 1.5×10^4^ cells/cm^2^ in (c) and (d). Figure a is adapted with permission from © 2010 Wiley Periodicals, Inc. J Biomed Mater Res Part A, 2010. Figure c is adapted with permission from © 2011 Wiley Periodicals, Inc. J Biomed Mater Res Part B: Appl Biomater, 2011. [2, 3]

A similar phenomenon is exhibited in Figure S4c and d. The results in Figure S4c is adapted from another publication of Yu et al [2, 3], using a similar fabrication process of TNTs and the autoclave method. But they used different cell types (mouse preosteoblasts MC3T3-E1 for Figure S4a and canine bone marrow stromal cells CBMSCs for Figure S4c) and cell density (normalized as 2.1×10^4^ cells/cm^2^ for Figure S4a and 1.6×10^4^ cells/cm^2^ for Figure S4c). Likewise, the predicted values are considerably lower than the experimental ones. But the prediction trend in Figure S4d is generally in line with Figure S4c when comparing TNTs with diameters of 30, 70, and 120 nm. Those two predictions prove that the GBDT model can learn the overall relations between cell proliferation and experimental variables.

It is worth mentioning that a drop could also be observed at 100 nm in Figure S4d, consistent with Figure S4b. Interestingly, a false conclusion might be drawn if we only analyze the most popular studied TNTs (30, 50, 70, 100 nm in the rectangle in Figures S4b and d), i.e., TNTs with a diameter size of 70 nm possess the highest cell proliferation. It suggests that the proliferation results are strongly influenced by the random choices of TNTs, partially explaining the discrepancy in the vast literature.

Similar to the analysis of increasing trends of cell proliferation over nanotube diameter, we conduct targeted model predictions on the decreasing trends from the literature. Figures S5a and c are adapted from two research groups, showing that cell proliferation deteriorates monotonously with the rise of nanotube diameter [4, 5]. The predictions in Figures S5b and d are not gratifying enough; however, both forecasts show that cell proliferation declines with nanotube diameter, in line with the experimental findings in Figures S5a and b, respectively.

Figure S5e is adapted from the same publication as Figure S5c. However, a higher cell density (compare 1.4×10^4^ cells/cm^2^ in Figure S5e to 2.8×10^3^ cells/cm^2^ in Figure S5c) is utilized to evaluate how cell density affects cell proliferation. On day 1, there is a slight decrease with the enlargement of nanotube diameter; however, this decline vanishes on day 2, suggesting higher cell density can diminish the differences among different nanotubes. It further confirms the high feature importance of cell density on cell proliferation (Figure 2b). The corresponding prediction trend of Figure S5e in Figure S5f shows a relatively similar steady trend for nanotubes with diameters smaller than 100 nm, with deterioration on 100 nm TNTs. The decline can be ascribed to the relatively low proliferation data for 100 nm nanotubes in our dataset, and the model forecasts based on the collected dataset.

Comparing the experimental variables in Figure S4 to those in Figure S5, cell density indeed plays a crucial role in cell proliferation on TNTs with varied dimensions, while annealing and autoclaving have been used for both increasing (Figure S4) and decreasing (Figure S5) trends. Compare cell density in Figure S4 to Figure S5, a high cell density is required for the increasing trend (Figure S4), and increased cell density can tune cell proliferation trend from decreasing to smooth (Figures S5e and f).

**
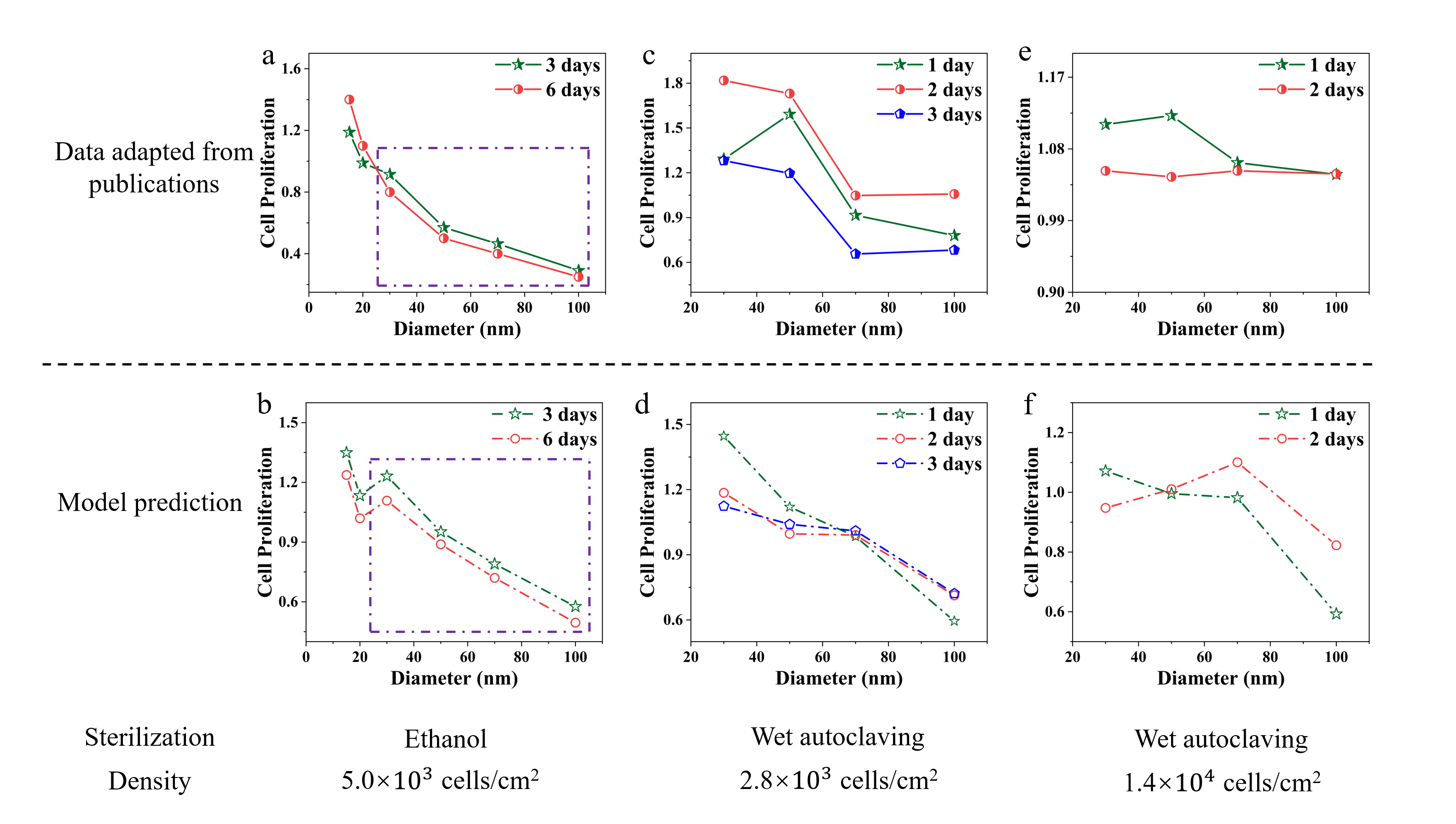
 Fig. S5.** Comparison between measured values (a, c and e) and corresponding model predictions (b, d, and f). All samples are annealed. Figure a is adapted with permission from © 2007 American Chemical Society, Nano Lett, 2007. Figure c and e are adapted with permission from © 2011 Elsevier B.V, Mater Sci Eng C, 2011 [4, 5].


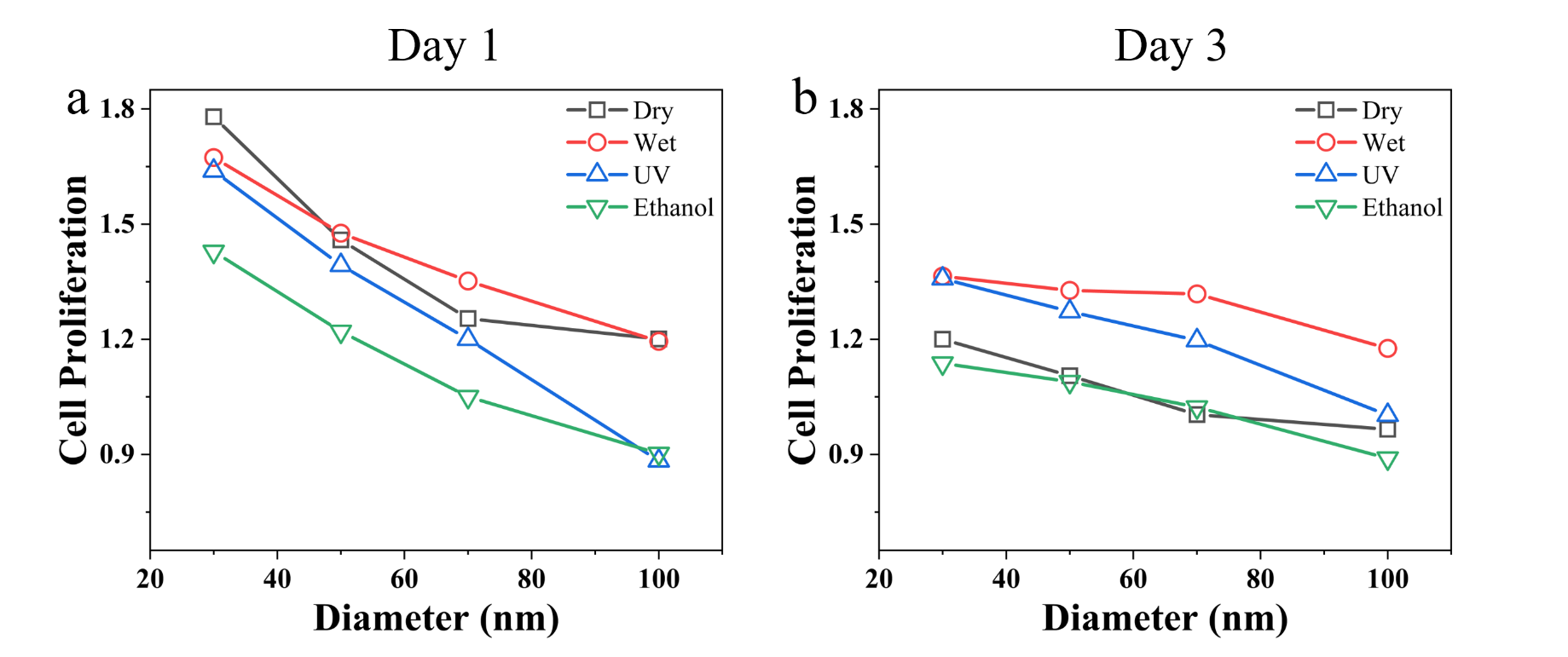


**Fig. S6.** GBDT model predicted cell proliferation trends with varied sterilization methods. All TNTs are annealed and cell density is 1.0×${10}^{4}$ cell/cm^2^.


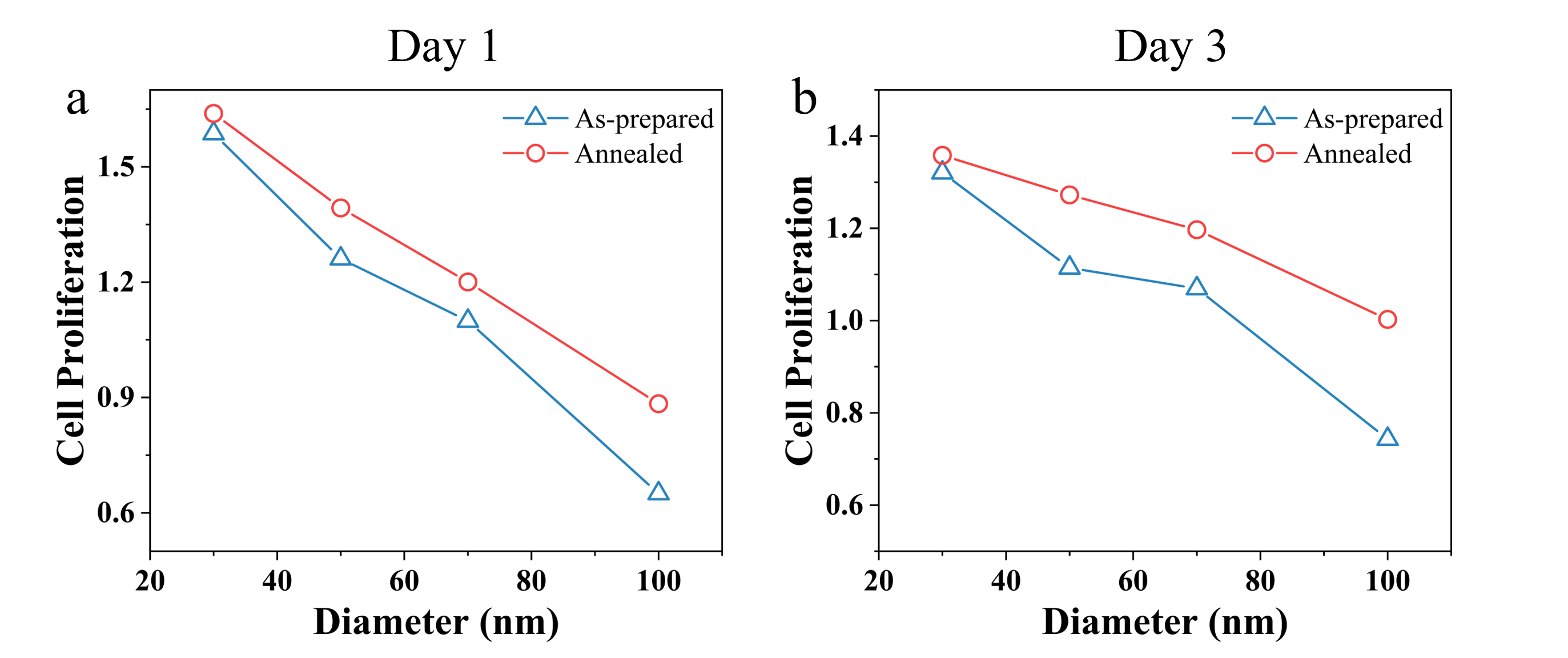


**Fig. S7.** GBDT model predicted cell proliferation trends with varied crystalline forms. Cell density is 1.0×${10}^{4}$ cell/cm^2^ and sterilization method is UV irradiation.


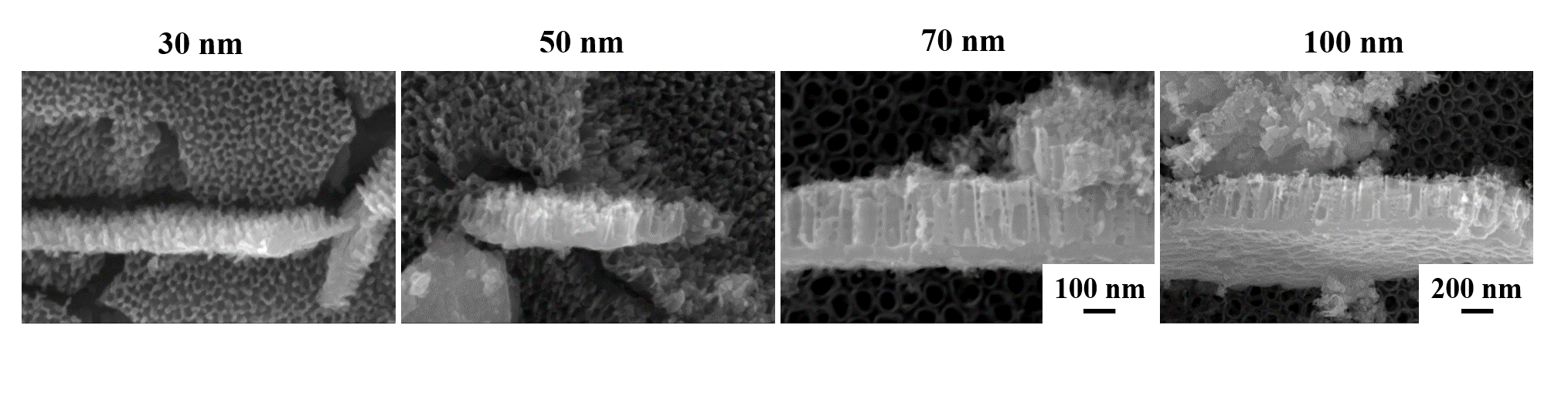


**Fig. S8.** The cross-sectional SEM views of the UV-irradiated TNTs.





**Fig. S9.** Nanotube wall thickness varied with tube diameter and sterilization methods.


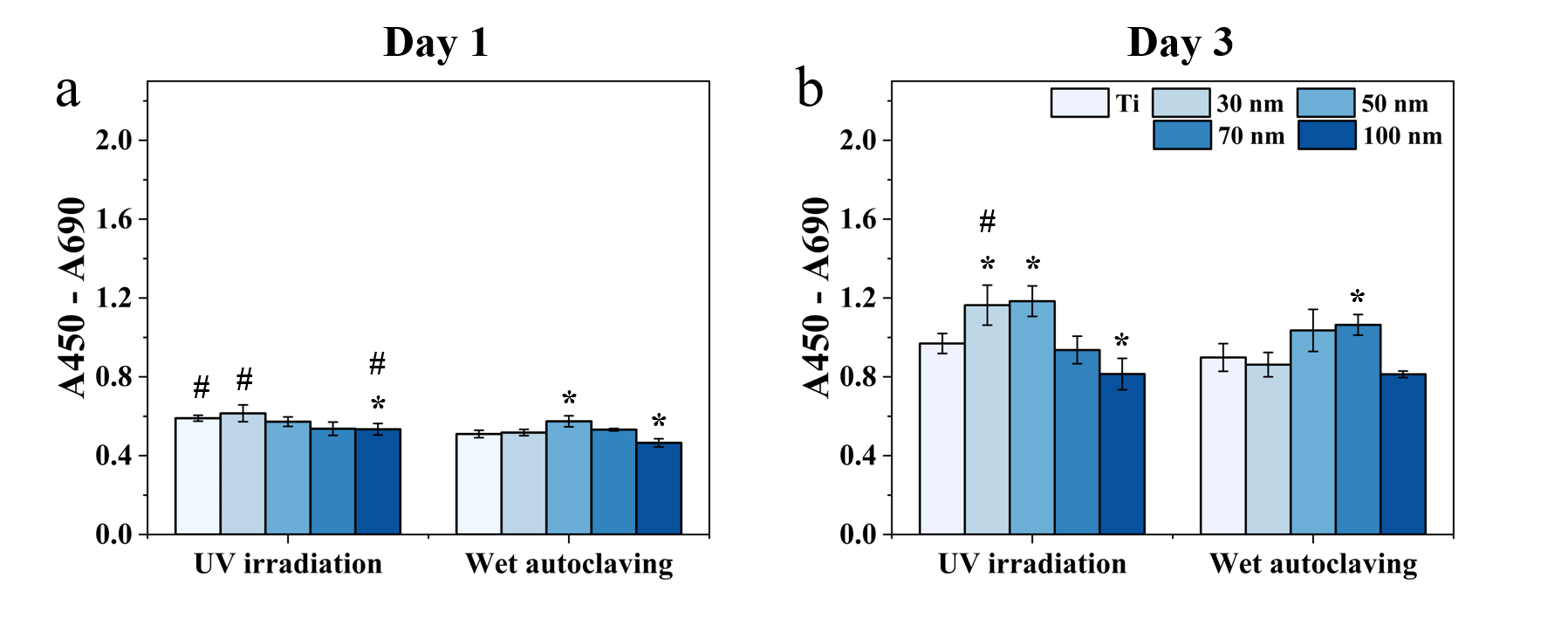


**Fig. S10.** The WST-1 cell viability results on annealed TNTs after 1 day (a) and 3 days (b) culture. Unmodified titanium foils are used as a control group. * p < 0.05 indicates a significant difference comparing to titanium, and # p < 0.05 indicates a significant difference between UV irradiated and wet autoclaved samples.

# References

[1] Fabian P, Gaël V, Alexandre G, et al. Scikit-learn: Machine Learning in Python. Journal of Machine Learning Research 2011;12:2825-30

[2] Yu W, Jiang X, Zhang F, et al. The effect of anatase TiO_2_ nanotube layers on MC3T3-E1 preosteoblast adhesion, proliferation, and differentiation. J. Biomed. Mater. Res. Part A 2010;1012-22

[3] Yu W, Jiang X, Xu L. Osteogenic gene expression of canine bone marrow stromal cell and bacterial adhesion on titanium with different nanotubes. J. Biomed. Mater. Res. Part B 2011;99B:207-16

[4] Park J, Bauer S, Mark K. Nanosize and vitality: TiO_2_ nanotube diameter directs cell fate. Nano Lett. 2007;7:‏1686-91

[5] Oh S, Brammer KS, Moon KS, et al. Influence of sterilization methods on cell behavior and functionality of osteoblasts cultured on TiO_2_ nanotubes. Mater. Sci. Eng. C. 2011;31:873-9
